# The Effects of Layout Order on Interface Complexity: An Eye-Tracking Study for Dashboard Design

**DOI:** 10.3390/s24185966

**Published:** 2024-09-14

**Authors:** Nuowen Zhang, Jing Zhang, Shangsong Jiang, Weijia Ge

**Affiliations:** College of Furnishings and Industrial Design, Nanjing Forestry University, Nanjing 210037, China; katherinaz@126.com (N.Z.); shangsongjiang@njfu.edu.cn (S.J.); geweijia@njfu.edu.cn (W.G.)

**Keywords:** human–computer interface, eye-tracking, dashboard, interface complexity, layout order

## Abstract

This study investigated the effect of layout order on the complexity of the dashboard interface based on screen-based eye trackers. By simplifying and abstracting dashboard interfaces and incorporating subjective ratings (symmetry and unity calculations), we successfully manipulated the levels of complexity and layout order of the interface materials. Using four types of eye movement data (total fixation count, total gaze duration, scanning paths, and hotspot maps) and behavioral data, we compared participants’ visual search behavior on interfaces with different layout orders and complexity levels. Experiment 1 revealed a significant interaction between layout order and interface complexity, with participants performing significantly better in the high-level layout order condition. Experiment 2 confirmed that the position of the core chart plays a crucial role in users’ visual search behavior and that the optimal layout order for the dashboard is to place the core chart on the left side of the interface’s horizontal axis, with partial symmetry in the no-core chart areas. This study highlights the effectiveness of eye-tracking techniques in user interface design research and provides valuable insights into optimizing dashboard interface design. Designers should adopt the design principle of “order is more” in addition to “less is more” and consider designing the core chart in the left-center position.

## 1. Introduction

With the continual advancement of computer technology, user interfaces have become increasingly functional and informative, incorporating a variety of visual elements such as maps, diagrams, system interfaces, monitoring screens, and information lists. A prime example is the dashboard, which typically features multiple well-organized charts and graphs. These organized visuals are structured based on the operational requirements of interface tasks, creating a specific visual hierarchy [1,2]. However, in practical applications, as system functions continue to evolve and expand, dashboards are becoming more complex, with an increasing number of charts and diagrams. This enriched layout often leads to overly complicated dashboard interfaces. It is widely recognized that human attention and cognitive resources are limited. As the number of charts in dashboard-style interfaces grows, users allocate fewer attentional resources to each element, resulting in less efficient visual processing [3,4]. Therefore, minimizing interface complexity without compromising functionality has emerged as a significant concern in dashboard interface design.

Current dashboard-type interface designs mainly use the conventional web interface design methods, such as checkerboard layout [5], horizontal layout [6] masonry layout [7], vertically integrated layout [8], and center layout [9]. For example, the center layout usually places the core chart (e.g., core maps) in the middle of the interface and then places non-core charts on both sides. The principles of layout design for user interfaces include narrative, analytical, and embedded layout [10]. However, those design approaches mainly focus only on the information categories [11], system functions [12], or task relevance [13], of the interface, rather than the overall visual order in the layout organization.

Consequently, the layout organization between charts in dashboard interfaces is not always regular and aligned. When the interface layout is less visually disorganized and logically structured from chart to chart, this interface layout seems to interfere with the user’s visual experience, as a disorganized interface layout seems more complex. However, previous studies on the relationship between interface complexity and layout have focused on structural properties, such as spacing [14], boundaries [15], and symmetrical form [6]. No prior research has investigated the impact of layout organization on the perception of interface complexity, and the influence of different visual orders in layout organization on cognition remains uncertain. We propose that the visual order and logical structure between charts in the layout organization should impact interface complexity.

We find these hypotheses plausible because studies in related fields have indicated that psychologically relevant organization, such as symmetry [16], structural variables [15], and grouping [7] in images and graphics, affects esthetic evaluation [17] and user experience [18]. For example, Gartus and Leder [19] observed that different types and orders of symmetry in pictures affect the accuracy of human observers in detecting radially symmetrical dot patterns. Fan et al. [16] found that participants indicated a better esthetic preference for Chinese ink paintings with local symmetry. More importantly, unlike the complexity perception process for images or graphics, the user’s cognitive flow in the dashboard interface is not random. The user’s perception of interface information and layout complexity is closely related to the cognitive flow, as the user needs to view the charts in a specific order according to the task. For example, users will typically view a core chart, such as a map, which usually occupies a large portion of the interface, first, and then they move on to other charts and view the core chart multiple times at once in the process. During this process, the user’s perception of interface complexity is influenced not only by the number of charts but also by the visual order in layout organization and the position of the core chart. Therefore, we propose the concept of “layout order”, which consists of the overall visual order and the sequential browsing order between charts. The overall visual order pertains to the visual balance (symmetry and unity) among charts in the interface layout, while the sequential browsing order is linked to the positioning of the core charts and the rationale behind the browsing sequence. It is important to note that, in practical application, not all dashboards are designed with the core chart positioned at the center of the interface; therefore, determining an optimal placement for the core image remains inconclusive.

Here, we introduce a screen-based remote eye tracker to further analyze the pattern (time and space) of eye movements in users’ visual browsing behavior on the dashboard interface. Previous studies have shown that eye-tracking techniques can provide a range of quantitative data on eye movement control, which can be utilized in user experience evaluation [20] and thus the technique is used as a guide in human–machine interface (HMI) design [21,22,23]. When users search for information on an interface, their eye movements objectively indicate attention distribution and information processing [24]. Using eye-tracking metrics such as total fixation count (TFC), total gaze duration (TGD), and scanning paths, users’ information processing and decision-making processes can be better visualized [25,26], thereby revealing visual search behaviors across different layout orders and interface complexities.

The aim of this study was to investigate the relationship between interface complexity and layout order in dashboard design and whether the layout order and the position of the core chart affect the user’s visual search behavior. The idea that highly organized forms of layout order can reduce the adverse effects of interface complexity and that the position of the core chart is crucial in layout design has not been experimentally tested before. We argue that layout order moderates interface complexity, and the level of layout order depends on how these charts are organized and where the core chart is located.

To explore this prediction, we experimentally manipulated the layout order and the position of the core chart in interface stimuli according to the layout forms in the actual dashboard interface. This study consisted of two phases: the first phase was a pre-experiment aimed at controlling the complexity and layout order of the interface stimuli in order to eliminate potential distractors. The first phase consisted of three processes: simplified abstraction of dashboard interfaces, subjective ratings, and symmetry and unity calculation. In the second phase, we conducted two experiments on the relationship between interface complexity and layout order in dashboard design. Experiment 1 explored how layout order affects interface complexity, while Experiment 2 further explored the optimal layout order and the best position of the core chart. In this study, a within-subject design was employed in the experiments, in which the two independent variables were layout order and complexity. Experiment 1 used a 2 (layout order levels) × 3 (complexity levels) within-subject design and Experiment 2 used a 3 (layout order levels) × 3 (complexity levels) within-subject design. The dependent variables of the experiment included behavioral data and eye movement data. The behavioral data included response time (RT), and the eye movement data included total fixation count (TFC) and total gaze duration (TGD). Therefore, compared to the quasi-experimental design used in previous related studies, this paper provides more evidence through an eye-tracking study, which is essential for examining the effect of layout order on interface complexity.

## 2. Related Work

While it is well known that interface information is more accessible to perceive when fewer charts are included and the interface is less complex, it is unknown whether a better layout order can moderate interface complexity. Several studies have mentioned a balance between visual order and complexity in esthetic appreciation [27,28,29], but most of these studies have been conducted on simple materials such as patterns [30] and images [31]. These previous studies have tended to define complexity and order as two separate absolute quantities [32] or to categorize order as the antonym of complexity [17,33]. However, they have not explored the correlation between interface complexity and layout order, especially using a screen-based eye tracker to study dashboard interfaces containing multiple infographics.

Eye-tracking technology, which non-invasively and precisely measures eye movements, is widely used to record and measure various cognitive processes, with a focus on visual cognition [34,35,36]. Numerous studies have explored eye-tracking technology across fields such as the visual system, psychology, and neurology [37,38]. Some key eye-tracking metrics such as total fixation count (TFC), total gaze duration (TGD), and scanning paths are important for evaluating interface layout studies. For example, fixation duration has been identified as a key metric for understanding how interface element layouts impact recognition efficiency [39]. Scanning paths can illustrate individual participants’ eye movement patterns during an experiment or the eye activity of multiple participants over a short period. The larger the focus radius, the longer the fixation time, indicating greater attention allocated by participants to stimuli in the focus area [40]. The hot spot map reveals visual behavior primarily by indicating the degree of attention to different areas of the interface through color variations [41,42], as red indicates areas that received more attention, while green indicates areas with less attention [43]. Based on these eye movement metrics, many scholars have used eye movement metrics to evaluate interface design. For example, Guo et al. reported that higher layout complexity leads to an increased number of saccades, while fixation count initially decreases before rising again [44]. Goldberg and Kotval found that well-structured functional grouping within layouts results in shorter scanning paths [45]. These research methods can help us explain the reasons why layout and order have an impact on visual search efficiency through eye movement data.

Visual complexity has long been recognized as a major factor interfering with users’ information perception, memorability [46,47], recognition thresholds [48,49], and esthetic preferences [1,50] and was commonly defined by the number of features and components. In the field of human–computer interaction, researchers have explored the adverse effects of complex interfaces in various types of user interfaces [11,44,51,52]. These include interfaces for nuclear power plants [53], computerized numerical control systems [54], geographic information systems [55], and in-vehicle information systems [5]. For example, Tang et al. [53] proposed that interface complexity significantly impacts operator decision-making in nuclear power plant interfaces, while Kang and Seong [56] observed that the complexity of the information system interface would affect the operator’s mental load. Researchers have proposed various factors to control or assess the complexity of interfaces, including entropy [31], texture clutter [57], compression rate [58], and task complexity [51]. Despite variations in the measurement of interface complexity, most current research regards interface complexity as a visually objective factor influenced by the number of interface elements. This includes the absolute number of interface features and components, which are counted or categorized according to the number of interface elements [50,59]. As a result, most designers adhere to the design principle of “less is more” in order to simplify the interface by reducing the number of elements such as graphics, colors, tables, text, icons, lists, and gauges.

However, recent empirical studies suggested that complexity is not solely based on the number of visual elements but is also influenced by factors such as familiarization [60], self-similarity [61], anisotropy [62], and stimulus type [63]. For example, our prior study [64] demonstrated that familiarity reduces perceived complexity, as prior knowledge modulates the effects of complexity. Wagemans [65] noted that the degree of combination of image-based figures affects complexity. These results suggest that the interface complexity of the dashboard is a relative, multidimensional property rather than a one-dimensional property. However, it is unclear whether the user’s cognitive difficulty is not only influenced by the complexity of the interface, but also closely related to the overall layout of the interface and the position of the core chart.

## 3. Research Methods

**H1:**  *The higher the layout order of the dashboard interface, the more efficient the user’s visual behavior and visual search will be.*

**H2:**  *There is an interaction between interface complexity and layout order, where high-level layout order can mitigate the adverse effects of high complexity on visual search performance.*

**H3:**  *Layout order consists of the overall visual order and the location of the core chart, as a better core chart position leads to a more organized visual browsing paths and shorter gaze duration.*

**Figure 1:**
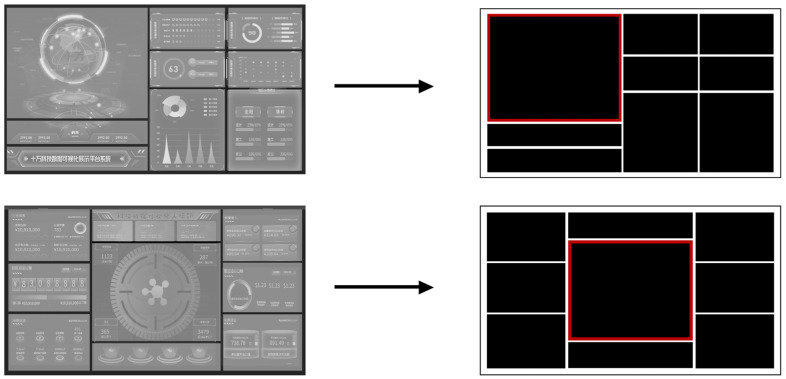
Examples of the process of dashboard interface abstracting into experimental material.

To confirm these hypotheses, we manipulated the complexity, layout order, and task target in our experiments and conducted two eye-tracking experiments specific to the study. We used the same material design approach described by Li et al. [66] with some modifications described below. Considering the reasonableness and applicability of the research object, we selected 60 typical interfaces from 200 actual used dashboard interfaces and 80 icons derived from these original interfaces. To reduce the potential effect of color on participants’ performance, a monochromatic color scheme was used for all stimuli: black (Hex: 000000) for charts and white (Hex: FFFFFF) for the background. By analyzing the formal functional descriptions and operational tasks of each interface, we further identified the location of the core charts within each interface. Since the core chart was the first to be noticed in the interface layout and was usually designed to be the most significant part of the interface, we highlighted the border of the core chart in red to distinguish it from the other charts (Figure 1). The experiment used a visual search paradigm where information in the charts was replaced with icons as search targets.

Subsequently, we recruited 50 volunteer participants with experience of using digital interfaces through the Nanjing Forestry University undergraduate community to rate the visual complexity and layout order of 60 typical dashboard interfaces and to rate the 80 icons (task target) on three factors: icon complexity, icon specificity, and icon familiarity. All ratings were conducted using a five-point Likert scale. For visual complexity ratings, interfaces were considered complex if they contained many elements (1 = definitely simple and 5 = definitely complex). For layout order ratings, interfaces were defined as the organizational regularity of the elements within the interface based on NGO’s esthetic evaluation [17] and Gestalt theory [67] (1 = definitely disordered and 5 = definitely ordered). The instructions for icons were the same as in previous studies [68,69]. For complexity ratings, symbols were considered complex if they contained many elements (1 = definitely simple and 5 = definitely complex). For order ratings, symbols were considered ordered if their elements were well organized or structured (1 = definitely disordered and 5 = definitely ordered). For concreteness ratings, graphical symbols were considered concrete if they were depicted like real items (1 = definitely abstract and 5 = definitely concrete). For familiarity ratings, symbols were considered familiar if they resembled an object that frequently appeared in daily life (1 = definitely unfamiliar and 5 = definitely familiar).

Based on the rating results, we observed that low-complexity interfaces contained fewer than 5 charts (complexity rating below 1.60), medium-complexity interfaces contained 7–9 charts (complexity scores between 2.30 and 2.72), and high-complexity interfaces contained over 12 charts (complexity scores above 4.33). For the layout order, low-level layout order interfaces (rating results below 1.64) were asymmetrical and irregular, with the core chart not centered or aligned on any axis. Mid-level layout order interfaces (rating results between 2.21 and 2.68) were partially symmetrical, with the core chart on the horizontal or vertical axis but not centered. High-level layout order interfaces were fully symmetrical, with the core chart in the center (rating results above 4.32 or more). Specifically, since the size of the core chart was generally more significant than other no-core charts, we found that the position of the core chart was closely linked to the degree of symmetry of the interface; for example, the interface was completely symmetrical only when the core chart was centered. Accordingly, we used the position of the core chart as one of the layout order definitions.

Subsequently, we categorized the experimental materials into low, medium, and high levels of interface complexity based on the number of charts: 5, 9, and 13. We then generated a total of 15 interfaces for each level, resulting in a total of 36 interfaces. For layout order, we defined it as the degree of visual symmetry and unity within the layout form of internal elements such as charts in the dashboard interface. We standardized and re-generated 36 interface stimuli with three levels of layout order: 12 high-level layout order interfaces with visually symmetrical and uniform layouts, placing the core chart in the center of the interface; 12 mid-level layout order interfaces with partially symmetrical and uniform layouts, positioning the core chart (1920 × 1080 pixels) along either the horizontal or vertical axis of the interface; and 12 low-level layout order interfaces with asymmetrical, unevenly distributed, and inconsistent layouts; here, the core chart is not aligned with any axis on the interface. To prevent interference caused by position randomness within mid-level and low-level layout order interfaces, we placed the core chart at one quadrant according to four positions: top, bottom, left, and right.

Although previous studies have not experimentally manipulated interface order, the visual symmetry, balanced distribution, and visual unity were computable and related to the esthetic evaluation [17,70] in the proposed layout order. To validate our layout order definition, we selected the two most relevant evaluation models from the esthetic model proposed by NGO [17], symmetry and unity, and then calculated these for all interfaces. Notably, considering the characteristics of the dashboard interface and users’ visual browsing habits, we selected only two metrics from the symmetry model: vertical and horizontal symmetry. The formulas are depicted in Table 1. The programs were written in Python, and the results are indicated in Table 2. Subsequently, we analyzed the results of the calculation for the three indicators for the 45 interfaces using analysis of variances (ANOVAs), followed by Newman–Keuls comparisons. The results indicated that there were significantly different in unity (F (2, 33) = 46.11, *p* < 0.001) and symmetry (F (2, 33) = 235.4, *p* < 0.001) ratings among the high-level, mid-level, and low-level layout order interfaces. The results confirmed that our definition of layout order was reliable.

To exclude the effect of icon complexity on participants’ cognition, we selected 20 icons with medium complexity ratings (mean = 2.46, standard deviation (SD) = 0.14), high concreteness (mean = 4.22, SD = 0.22), and high familiarity (mean = 4.56, SD = 0.24). The ANOVA results revealed no significant difference in concreteness (F(19,980) = 0.027, *p* = 0.871), complexity (F(19,980) = 0.039, *p* = 0.845), or familiarity (F(19,980) = 0.063, *p* = 0.805). All these icons were placed in the center of the information charts. The icon size was standardized at 48 × 48 pixels, and the color was all designed to be gray (Hex: 505050) to mitigate the potential effect.

**Figure 2:**
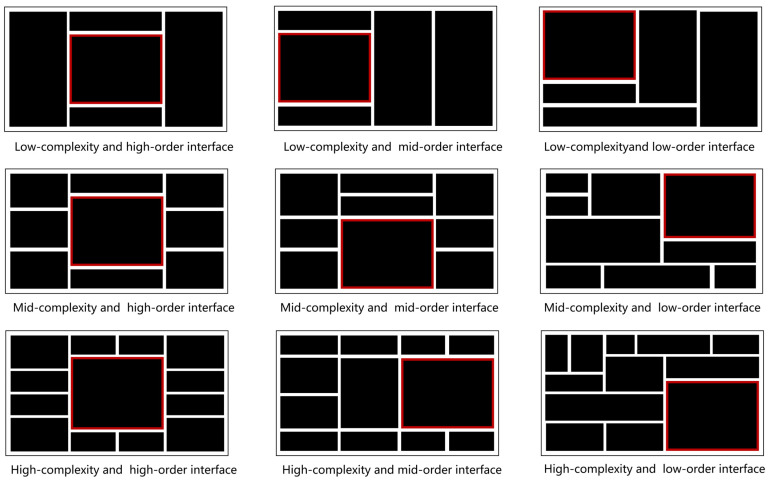
Examples of the experimental material on three different complexity levels and three different layout order levels.

Using the simplified abstraction of dashboard interfaces, subjective ratings, and symmetry and unity calculations, we successfully manipulated the levels of complexity and layout order of the interface materials, all of which were novel to the participants, thus eliminating potential confounding factors. The examples are shown in Figure 2.

## 4. Research Content and Results

This research involved two eye movement experiments. The purpose of Experiment 1 was to explore the effects of layout order on interface complexity and whether the high-level layout order improved users’ visual behaviors. Specifically, we aimed to compare users’ visual search behavior under two levels of layout order (high and low) and three levels of interface complexity (high, medium, and low) to verify whether there was an interaction between complexity and order and whether high-level layout order could mitigate the interference effect of complexity. The purpose of Experiment 2 was to analyze the impact of different positions of the core chart in layout order on user behavior and determine the optimal position for the core chart in the interface.

All experiments in this study were approved by the International Review Board of Nanjing Forestry University. Participants were informed that their experimental data would be recorded and analyzed for a scientific research project, and they signed a consent form before participating in the experiment.

### 4.1. Experiment 1: The Effects of Layout Order on Interface Complexity

#### 4.1.1. Participants

Forty-three participants (with ages ranging from 20 to 25; mean = 22.3,) with experience of using digital interfaces, recruited from Nanjing Forestry University undergraduate community, participated in Experiment 1, including seventeen males and twenty-six females. Participants received a payment between CNY 15 and CNY 20, depending on their performance (accuracy) on the task. All subjects were right-handed with normal or corrected-to-normal vision and no color blindness or color weakness.

#### 4.1.2. Design and Materials

**Figure 3:**
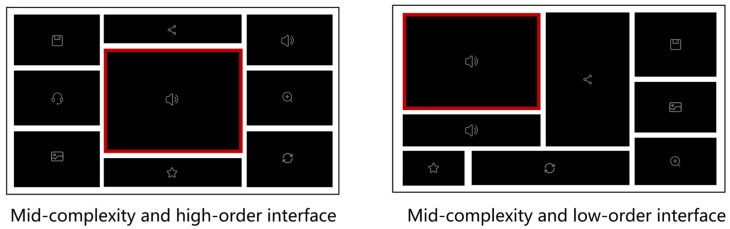
Examples of a mid-complexity and high-order interface and a mid-complexity and low-order interface in Experiment 1.

The experiment used a 2 (layout order levels) × 3 (complexity levels) within-subjects design, with each participant exposed to all six interface types: (low-complexity and low-order), (low-complexity and high-order), (mid-complexity and low-order), (mid-complexity and high-order), (high-complexity and low-order), and (high-complexity and high-order). Based on material design in research methods, low, medium, and high interface complexity was defined regarding the number of charts (5, 9, and 13), while layout order was determined by the degree of visual symmetry, balanced distribution, and visual uniformity of the chart layout forms within the interface. From the 36 standardized interface stimuli generated previously, we selected 4 interfaces for each of the 6 interface types, totaling 24 interface stimuli. The location of the core chart was closely linked to the degree of symmetry in layout order. Specifically, the core charts of all high-order interfaces were positioned in the center of the interface, while the core charts of all low-order interfaces were located in one of the four quadrants. Figure 3 displays examples of (mid-complexity and high-order) interfaces and (mid-complexity and low-order) interfaces.

This experiment used the multi-chart area outside the core chart (red-bordered rectangle) in each interface as the area of interest to analyze the eye movement data. The Tobii 250 Fusion Eye Tracker (a screen-based remote eye tracker with a sampling rate of 250 Hz and an accuracy of 0.9) was used for the eye-tracking activity, and the eye movement data and reaction times were collected with ErgoLAB. The experiment was conducted on an HP 27-inch monitor with a resolution of 1920 × 1080 pixels with a black screen background. The participants were seated 580–620 mm away from the central displays.

#### 4.1.3. Procedure

Participants performed a visual search task during two sessions, each consisting of 24 trials. There was a 5 min break between the sessions. Each experimental trial began with a fixation indicated, and the participants had to press any button to continue. Then, the participants observed a randomly selected icon in the center of the screen for 1000 ms, followed by an interface stimulus of six interface types. The participants were required to view all charts containing icons (48 × 48 pixels) and count the total number of appearances of the target icon. There was no time limit to respond, but the participants had to focus and respond quickly to the best of their ability. To avoid downward scanning while searching for the corresponding key on the keyboard, which would affect the eye movement results, the participant pressed the space bar and entered the numerical answer on the next screen. The target icons in each interface were randomly set to 1, 2, or 3. At the trial’s end, a blank screen was presented for 1000 ms before the next trial.

**Figure 4:**
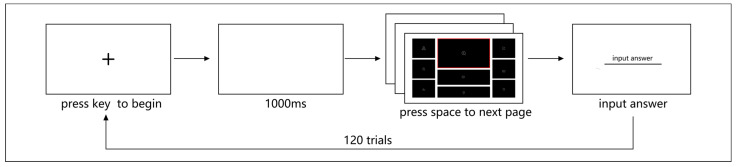
Flow chart of Experiment 1.

There was a practice experiment including four trials before the formal experiment. The experimental instructions were presented to the participants via the monitor to ensure that the participants understood the experimental procedures. Then, each participant performed an eye calibration through the eye tracker and started the practice experiment. After completing the practice experiment, the participants rested for 30 s and then started the formal experiment. The entire experiment lasted approximately 25 min, as the experimental flow chart in Figure 4 shows. All experiments were conducted in the Human–Computer Interaction Laboratory of Nanjing Forestry University under normal office lighting (~300 lx).

#### 4.1.4. Data Analysis

A power analysis was conducted using the “pwr package” in R-studio [71,72] to ensure the sample size achieved adequate power. Since every participant underwent 60 trials in two sessions, we assumed a large effect size (η_p_^2^ = 0.14 [73]). The achieved power was 0.985, with α = 0.05.

The experimental results included behavioral data and eye movement data. The behavioral data included RT, and the eye movement data included total fixation count (TFC), total gaze duration (TGD), scanning path, and hotspot map. Both the behavioral data and eye movement data were recorded and exported by ErgoLAB. TFC was the total count of fixations in the AOI after completing a visual search task, reflecting the efficiency of the task, while TGD was the sum of gaze duration within each piece of information, reflecting the difficulty of the visual search task [74]. The hotspot map was primarily utilized to depict the browsing and gazing behavior towards the stimulus material. The color red indicated the most concentrated browsing and gazing, while yellow and green represented areas with less attention [75]. The scanning path explained the location and order of the fixation points on the stimuli and reflected the eye movement path of the participants as they viewed the dashboard interface [23]. The behavioral and eye movement data were processed and analyzed by R-studio. For the RT analyses, we considered only correct trials (3.3% error for the experiment). We excluded cases with RTs over three median absolute deviations above or below the median RT (10.3%) from the analysis. One participant was dropped from the experiment because their performance was two SDs below the mean of the remaining participants, leading to forty-two participants. Both RT and eye movement data were analyzed using linear mixed-effects regressions [76,77]

**Figure 5:**
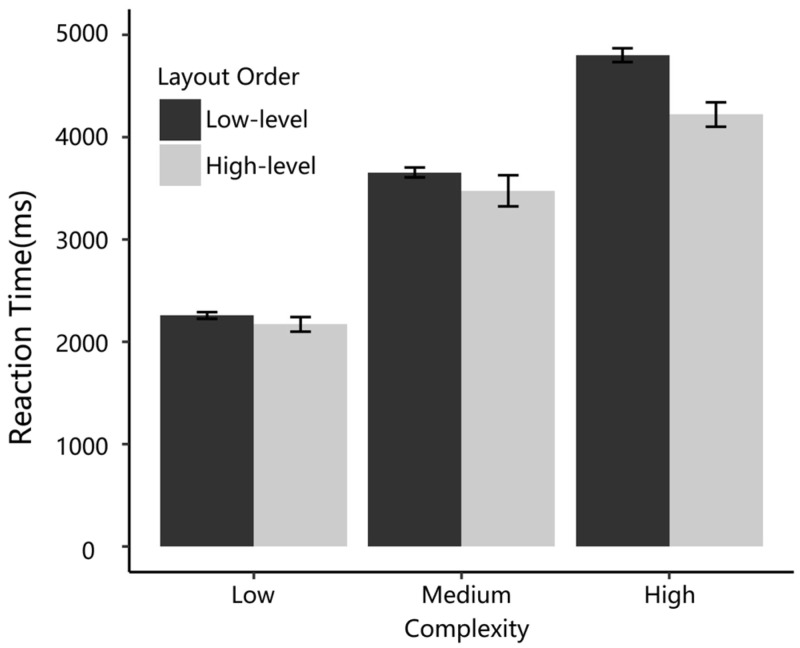
RTs of high-level and low-level order layouts at three different complexity levels.

Figure 5 displays the participants’ mean RT high-level and low-level orders in different complexity conditions. These participants indicated significant differences in various levels of interface complexity (ΔAIC = −1795.5, LLRχ2 (1) = 1799.52, and *p* < 0.001). The main effect of layout order was significant (ΔAIC = −25.7, LLRχ2 (1) = 27.72, and *p* < 0.001), such that the participants’ RT decreased from low-level to high-level layout orders. There was a significant interaction between order and complexity in the RT (ΔAIC = −16.5, LLRχ2 (1) = 20.53, and *p* < 0.001), such that as complexity increased, the difference in RT between highly ordered layout and low ordered layout increased, making the advantages of high-level layout order more apparent. These findings support H1 and H2.

The results of TFC and TGD are indicated in Table 3. As expected, the participants demonstrated significant differences in different levels of interface complexity in TFC (ΔAIC = −1208, LLRχ2 (1) = 1211.83, and *p* < 0.001) and in TGD (ΔAIC = −1096, LLRχ2 (1) = 1100.05, and *p* < 0.001). There was a significant difference between low-level and high-level layout orders in TFC (ΔAIC = 3, LLRχ2 (1) = 2, and *p* < 0.05) and in TGD (ΔAIC = 2.7, LLRχ2 (1) = 2, and *p* < 0.05), such that the participants indicated fewer fixations and shorter gaze durations in ordered conditions. This means that participants were more efficient and easier to find the target in high-level order conditions. Additionally, a significant interaction was observed between order condition and complexity levels in TFC (ΔAIC = −8, LLRχ2(1) = 12.379, and *p* < 0.001) and TGD (ΔAIC = −7.8, LLRχ2 (1) = 11.824, and *p* < 0.05), such that the participants’ eye movement behaviors varied with the level of interface complexity (performance was better when the complexity was higher). These results provided additional evidence beyond the RT results in support of H1 and H2: layout order affects visual behavior, as high-level order significantly contributes to the users’ visual behavior, and there was an interaction between complexity and layout order, with layout order mitigating the negative effects of complexity.

The example scanning path of the participants in high-level and low-level layout orders is indicated in Table 4, with different colors representing the different scanned paths. In both high-order and low-order interfaces, the eye movement paths of the participants initially focused on the target icon in the core chart. Then, they immediately shifted their attention to the no-core chart area after starting the search. While searching for the target icon, their attention moved within the perceptual span, causing a saccade gaze. In high-order interfaces, the participants’ eye movement started at the center of the interface and moved along the traditional reading directions (left-to-right and top-to-bottom). Since the layout in high-order interfaces was organized more regularly than in low-order interfaces, it led the eye more clearly to the AOI and made it easier to shift attention to the next icon. In contrast, the scanning paths in low-level order interfaces were more complex and randomized, consistent with the results in TFC and TGD.

**Figure 6:**
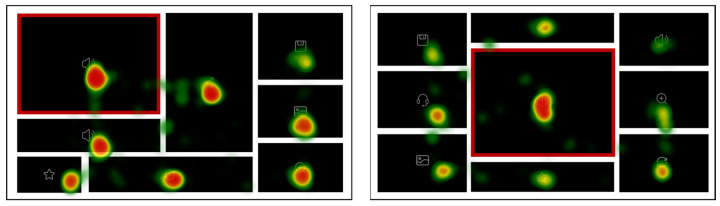
Hotspot maps of a mid-complexity and high-order interface and a mid-complexity and low-order interface in Experiment 1.

The example hotspot maps of typical high-level and low-level layout order interfaces are indicated in Figure 6. Since the icon in the core chart was the target, the core chart area was the most concentrated area of browsing and gazing. Compared with the high-level layout order interfaces, the hotspot values in the no-core chart area of the low-level order interface were significantly higher (more red areas), indicating that low-ordered layouts exhibited more views per element, and the participants were slower to find the target. The distribution of the users’ attention was more balanced and symmetrical in the high-level order interfaces, while the hotspots in low-level order interfaces were more scattered.

As anticipated, high-level order demonstrated improved search efficiency and had a positive impact on users’ visual behavior. The influence of complexity on participants’ visual performance was influenced by the layout order of the interface. As the complexity level increased, the impact of the order became more evident. The analysis of eye movement data provided further evidence to support H1 and H2, beyond the results obtained from reaction time (RT). Therefore, we argue that Experiment 1 supports the notion that layout order has a mitigating effect on complexity.

Furthermore, we concluded that the position of the core chart affects visual search behavior and that a high-level layout order that was completely symmetrical and exhibited the core chart in the center of the interface was the optimal layout format. However, since the layout order we defined involves three locations of the core chart, Experiment 1 only compared the high-order layout (with the core chart in the center) and low-order layout (with the core chart on neither the horizontal nor the vertical axis) without examining the specific effects of the mid-level layout order (partially visually symmetrical and partially visually uniform layouts, with the core chart located on either the horizontal or the vertical axis of the interface). To explore the optimal layout order and the best position of the core chart, we designed Experiment 2 for further validation.

### 4.2. Experiment 2: The Role of the Core Chart’s Position in the Layout Order

The purpose of Experiment 2 was to explore the optimal layout order and the best position of the core chart within the layout order and to explore whether H3 was valid. The experimental materials for low-level and high-level layout orders were identical to those in Experiment 1, and the mid-level layout order interface stimuli were selected from the standardized interface stimuli we generated previously. Since the high-order layout exhibited the core chart in the center of the interface and the mid-order layout exhibited the core chart located at four positions along the axis, we further compared the five positions of the core chart. Apart from adding mid-level layout order interface stimuli, Experiment 2 was the same as Experiment 1.

#### 4.2.1. Participants

Forty participants (with ages ranging from 21 to 26; mean = 23.9) with experience of using digital interfaces, recruited from Nanjing Forestry University undergraduate community, participated in Experiment 2, including seventeen males and twenty-three females. Participants received a payment between CNY 15 and CNY 20, depending on their performance (accuracy) on the task. All subjects were right-handed with normal or corrected-to-normal vision and no color blindness or color weakness.

#### 4.2.2. Experiment Material

**Figure 7:**
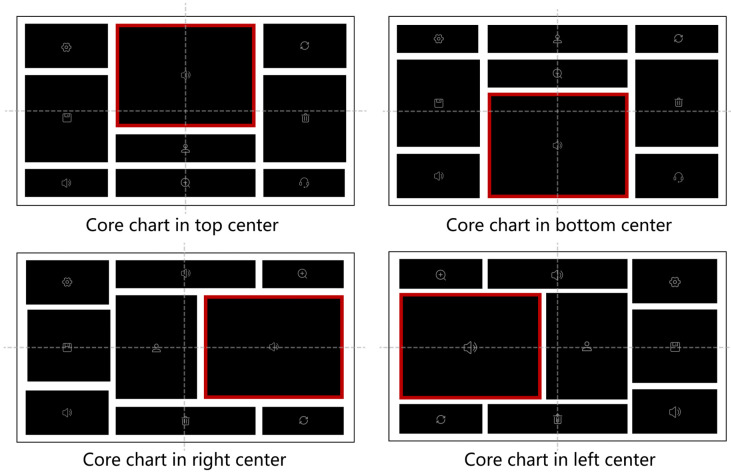
Examples of mid-complexity and mid-order interfaces with the core charts in four positions in Experiment 2.

Experiment 2 used a 3 (layout order levels) × 3 (complexity levels) within-subjects design such that each participant was exposed to all nine interface types: (low-complexity and low-order), (low-complexity and mid-order), (low-complexity and high-order), (mid-complexity and low-order), (mid-complexity and mid-order), (mid-complexity and high-order), (high-complexity and low-order), (high-complexity and mid-order), and (high-complexity and high-order). As in Experiment 1, interface complexity was defined regarding the number of charts (5, 9, and 13). We selected four interfaces for the nine interface types for thirty-six interface stimuli. The high-order and low-order interfaces were the same as in Experiment 1, and the mid-order layout order was added to the layout order factor. According to our definition in research methods, the center of the core chart in each mid-level layout order interface was located on the horizontal or vertical axis, and the no-core charts were partially symmetrical. To avoid interference caused by the random position of the core chart, we standardized the position and occurrence of the core charts on the axis. There were five locations of the core charts in the top center, bottom center, left center, and right center of the interface (in the mid-order layout, Figure 7), and mid-center (in the high-order layout, Figure 7). Given that the position of the core chart was closely linked to the level of layout order, high-order interfaces were visually perfectly symmetrical and uniform, mid-order interfaces were partially visually symmetrical and partially visually uniform, and low-order interfaces were visually asymmetrical, unevenly distributed, and inconsistent.

#### 4.2.3. Procedure

Experiment 2 followed the same procedure described in Experiment 1, with a few modifications noted. Participants performed a visual search task during two sessions, each consisting of 36 trials.

#### 4.2.4. Data Analysis

A power analysis was conducted using the “pwr package” in R-studio to ensure that the sample size achieved adequate power [71,72]. Since every participant underwent 72 trials in two sessions, we assumed a large effect size (η_p_^2^ = 0.14 [73]). The achieved power was 0.979, with α = 0.05 (η_p_^2^ = 0.14 [73]).

The experimental results included RT and eye movement data. For the RT analyses, we considered only correct trials (3.9% error for the experiment). Then, we excluded cases with RTs over three median absolute deviations above or below the median RT (5.5%). Both RT and eye movement data were analyzed using linear mixed-effects regressions [76,77].

**Figure 8:**
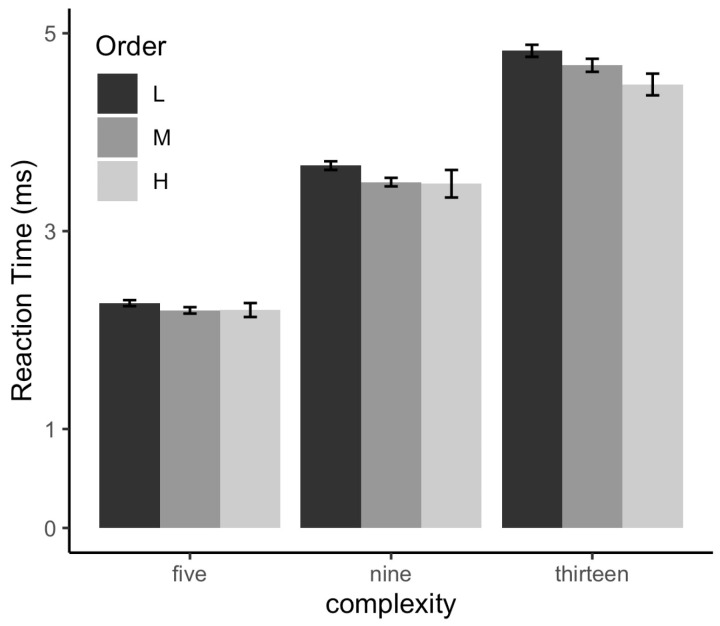
RTs of high-level layout order and mid-level layout order at three different complexity levels.

Figure 8 indicates the participants’ mean RT for high-level, mid-level, and low-level orders in different complexity conditions. As in Experiment 1, the participants indicated significant differences across the three levels of interface complexity (ΔAIC = −3249, LLRχ2 (1) = 3253.59, and *p* < 0.001), such that the participants’ RT increased from low-level complexity to high-level complexity. The main effect of layout order was significant (ΔAIC = −27, LLR χ2 (1) = 30.38, and *p* < 0.001), indicating that the participants’ RTs varied across the three levels of layout order. Importantly, the main effect of the core chart location was significant (ΔAIC = −29, LLR χ2 (1) = 35.286, and *p* < 0.001), and there was a significant interaction between the core chart location and complexity (ΔAIC = −1, LLR χ2 (1) = 12.84, and *p* = 0.045), such that the position of the core chart within the layout order affects the interface complexity.

The results of TFC and TGD were consistent with Experiment 1. There was a significant main effect of complexity in TFC (ΔAIC = −2512, LLR χ2 (1) = 2516.01, and *p* < 0.001). On TGD, ΔAIC = −2287.3, LLR χ2 (1) = 22291.30, and *p* < 0.001 indicated that participants identified the target more quickly in low-complexity conditions. The main effect of layout order was also significant for TFC (ΔAIC = −36, LLR χ2 (1) = 39.32, and *p* < 0.001) and TGD (ΔAIC = −14.9, LLR χ2 (1) = 18.88, and p < 0.001) such that the participants’ RT decreased from low-level to high-level layout orders.

In contrast to Experiment 1, there was no significant interaction between order and complexity for RT (ΔAIC = −18.3, LLR χ2 (1) = 6.4294, and *p* < 0.001), TFC (AIC = 6, LLR χ2 (1) = 2.68, *p* = 0.61), or TGD (AIC = 2.2, LLR χ2 (1) = 5.87, and *p* = 0.21). Given the addition of the mid-order level in Experiment 2, one possible explanation for these results was that the mid-level layout order exhibited a different effect on complexity due to the core chart changes in four positions. As we proposed in H3, the position of the core chart within the layout order affects the interface complexity. Therefore, we further compared and analyzed the RT and eye movement data for the five positions of the core chart in high-order and mid-order conditions.

**Figure 9:**
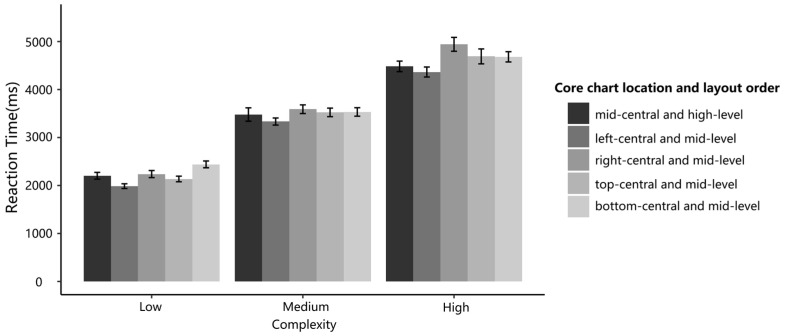
RTs of five different layout order level interfaces at three complexity levels.

Figure 9 plots the mean RT of the five positions in different complexity conditions. The participants indicated significant differences in three levels of interface complexity (ΔAIC = −732.2, LLRχ2 (1) = −736.19, and *p* < 0.001) and the five core chart positions (ΔAIC = −5.4, LLRχ2 (1) = −5.58, and *p* < 0.05). Additionally, the participants performed significantly faster when the core chart was in the left-central position of the interface than in the mid-central position (ΔAIC = −6.2, LLRχ2 (1) = 1.26, and *p* < 0.05). These results indicated that the advantages of high-level layout order also appeared in one mid-level order layout where the core chart was in the left center.

**Figure 10:**
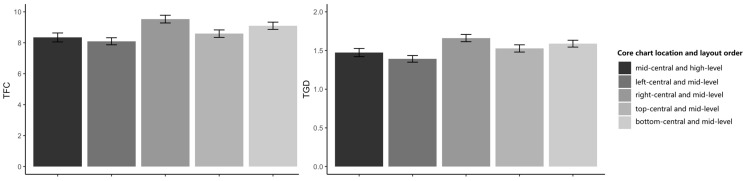
Eye movement data of five different layout order levels.

The results of TFC and TGD for the five locations are depicted in Figure 10. The main effect of interface complexity was significant for TFC (ΔAIC = −5537, LLRχ2 (1) = 557.67, and *p* < 0.001) and TGD (ΔAIC = −512.5, LLRχ2 (1) = 516.46, and *p* < 0.001). However, the main effect of core chart position was non-significant in TFC (ΔAIC = 1.6, LLRχ2 (1) = 0.49, and *p* = 0.48) and TGD (ΔAIC = −0.5, LLRχ2 (1) = 1.53, and *p* = 0.22). Additionally, there was no significant difference between the centered core chart layout and the left-centered core chart layout for TFC (ΔAIC = 3.6, LLRχ2 (1) = 0.41, and *p* = 0.82) and TGD (ΔAIC = 3.7, LLRχ2 (1) = 0.30, and *p* = 0.86).

Table 5 indicates the example scanning path of the participants in mid-central and high-order and left-central and mid-order interfaces, with different colors representing the different scanned paths. As in Experiment 1, the eye movement paths of the participants initially focused on the target icon in the core chart and then shifted to the area of no-core charts after starting the search. In the left-central and mid-order interfaces, the visual scanning path was clearer and more consistent, with the participants starting from the left and then navigating through the no-core charts area in a clockwise direction (from right-central to top, left to right, and top to bottom). This result revealed that when the core chart was in the left-central position and the area of no-core charts was partially symmetrical (mid-order layout), the participants’ attention shifted faster during visual search than in the high-order layout where the core chart was in the center, and the area of no-core charts was full symmetrical.

**Figure 11:**
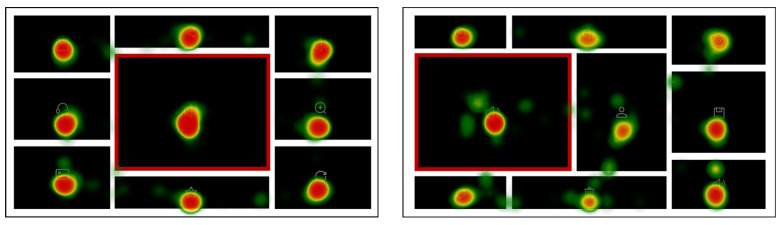
Hotspot maps of the high-level layout order interface and mid-level layout order interface.

The example hotspot map of typical mid-central and high-order and left-central and mid-order interfaces are indicated in Figure 11. Comparing the hotspot maps for the two core chart positions, the hotspots in the no-core chart area were noticeably redder in the mid-central and high-level layout order, while in the left-central and mid-level layout order, there were few red areas and more green areas. These results were consistent with the TFC and TGD findings and provided additional support for H3: the participants’ eye movement was affected by the core chart position.

In summary, Experiment 2 replicated the findings of Experiment 1 and provided support for H3, demonstrating that the positioning of the core chart within the layout order impacts interface complexity. Significantly, the participants exhibited faster performance in left-central and mid-order interfaces compared to mid-central and high-order interfaces. Thus, it was confirmed that the placement of core charts is a crucial aspect of the layout order, in addition to the overall visual arrangement.

## 5. General Discussion

In interface design, complexity has long been recognized as a significant factor affecting users’ information perception [44,47]. A dashboard is one of the typical information interfaces containing multiple well-partitioned charts. Unlike the complexity in images or graphics, interface complexity in a dashboard involves various visual charts and information forms. Most previous studies regard interface complexity as an objective factor influenced by the number of elements and components within the interface [1,78]. Consequently, to follow the design principle of “less is more”, most designers reduce the number of interface elements to minimize the effects of complexity. Although some studies on dashboard-style interfaces have proved the importance of position and size of each chart [79] and the usability of information analysis [11], these studies ignored the impact of the logical structure and layout organization between charts within an interface on complexity.

Our study investigated the relationship between interface complexity and layout order using eye-tracking technology and examined the effect of layout order on visual search behavior. Total fixation count (TFC), total gaze duration (TGD), and scanning path are key eye-tracking metrics that clearly reflect users’ visual search behavior across different interfaces, thereby assisting in optimizing interface design from a cognitive perspective. For example, objective eye-tracking metrics, such as fixation counts and durations, indicate that Mobile App interface layouts better align with the “F” visualization model, resulting in higher user satisfaction [39]. Guo et al. [44] reported that high-complexity interfaces cause participants to exhibit more revisits, fixations, and saccades. Goldberg and Kotval [45] found that well-organized functional grouping within computer interface layouts results in shorter scanning paths for participants. Based on previous eye-tracking experiment designs and analysis methods [44,80], we proved the interaction between interface complexity and layout order from behavioral data and eye movement data.

The current design methodology for dashboard-style interfaces is mainly based on information categories [11] and system functions [12], and therefore the interface layout of dashboard follows the principle of “form follows function”. Here, our study focused on the interaction between overall visual order, intrinsic layout organization and complexity. More importantly, the user’s cognitive flow in the dashboard interface is not random; users usually need to view a core chart firstly and then move on to other charts. For example, Hind [81] pointed out that the dashboard layout of a car interface is mainly determined by task relevance. Goudsmit et al. [13] proposed that dashboard design should first consider user requirements. Therefore, we proposed a visual order in layout organization consisting of the overall visual order and the sequential browsing order between charts, which we defined as “layout order”. One point to note is that the sequential browsing order between charts is not only related to the task, but also to the logical correlation between the core and no-core charts.

Through the simplified abstraction of dashboard interfaces, subjective ratings, and symmetry and unity calculations, we experimentally manipulated the levels of complexity and layout order of interface stimuli. Experiment 1 explored how layout order affects interface complexity. Based on four types of eye movement data, namely, TFC and TGD, scanning paths, hotspot maps, and RT, it was proven that there was a significant interaction between layout order and interface complexity, with participants performing significantly better in the high-level layout order condition. Experiment 2 further explored the optimal layout order and the best position of the core chart. The participants revealed faster performance in left-central and mid-order interfaces than in mid-central and high-order interfaces. The results confirmed that the effect of complexity is influenced not only by the organization of the charts but also by the position of the core charts within the interface.

While the study and evidence we presented are novel, our stance is consistent with some image-related research [2,82]. For example, Kemps [83] examined how visual complexity is affected by both quantifiable physical attributes and structural factors. Chipman [2] proposed that recognizing the psychologically relevant organizational and structural variables can reduce subjective complexity. However, existing studies on the relationship between order-related factors and complexity have primarily used quasi-experimental designs that relied on pre-existing differences in the order of the stimuli [44,45]. Compared with the stimuli used in previous studies, the current study employed a stricter experimental design. (a) We introduced the concept of layout order; experimentally manipulated the level of layout order through a simplified abstraction of dashboard interfaces, subjective ratings, and symmetry and unity calculations; and generated a series of interface materials. This design eliminated other factors affecting layout order and interface complexity, minimizing pre-existing differences in familiarity, complexity, and concreteness between target icons. (b) To better reflect the actual browsing order between charts within dashboard, we incorporated cognitive flow and emphasized the key role of the core chart in layout order. We placed the target icon in the core chart of each interface to ensure that the participants started with the core chart. (c) We optimized the experimental task by randomly displaying one to three targets in the interface instead of just one. This approach requires participants to navigate the entire interface to determine the total number of target icons, better simulating the actual task of using a dashboard interface. These manipulations enhance the validity of our measures and confirm that the moderating effect on complexity is due to layout order rather than other confounding factors.

One explanation for the advantages of high-level layout order results from the concept of perceptual grouping in Gestalt psychology [67]. According to Gestalt theory, visual recognition usually groups elements more quickly if the elements are symmetrical [2]. Since the organization of the parts in an object is crucial in visual perception, the visual system automatically organizes well-structured elements most simply and coherently rather than treating them as separate elements [84]. From this perspective, it seems reasonable to assume that visual processing is influenced by the degree to which the internal elements are organized in a rational and logical order. In our experiment, the layout order is related to the overall visual order and the sequential browsing order between charts. Since high-order layout interfaces use visually perfectly symmetrical and uniform layouts, the ordered layout organization interface is easier to group. Consequently, the no-core charts in high-level order interfaces can be grouped into fewer units faster, with lower cognitive load and shorter visual processing times, thus mitigating the effects of high complexity. In contrast, in low-complexity interfaces, where the number of no-core charts was inherently small, the limited number of groupings diminished the advantage of high order.

Another plausible explanation results from the chunking theory of Miller [85], Simon [86], and Reder et al. [60]. Chunking theory posits that when information can be encoded or chunked into higher-level units, it is easier to process and consumes fewer attentional resources [87,88]. Our prior study demonstrated that the strength of the chunks modulates the effects of complexity [67]. According to these views, we contend that less attention and fewer resources are required to process high-level order interfaces. Consequently, high-level order interfaces offer greater cognitive advantages because fewer attentional and working memory resources are consumed during the cognitive process of the ordered layout, and the chunking strength of the interface’s information subsequently becomes stronger. Contrarily, low-order layout interfaces have a weaker chunking strength due to the disordered layout organization. Furthermore, given that visual working memory (VWM) can store about four basic objects [89], and in low-complexity conditions, there were four no-core charts per interface, which does not exceed the VWM capacity, this could explain why the advantage of high-order was less apparent in low-complexity interfaces.

More importantly, our research challenges the common assumption that “a fully symmetrical interface with the core chart in the center is the optimal interface layout” [9]. While this view is also reflected in our results of subjective ratings and symmetry and unity calculations, the reaction time data and eye movement data from Experiment 2 indicated that when the core chart was in the left-central position and the area of no-core charts was partially symmetrical (mid-order layout), the participants’ attention shifted faster during visual search than in high-order layout where the core chart was in the center and the area of no-core charts was full symmetrical. We contend that the difference is due to the position of the core chart and the browsing order, since the participants initially focused on the target icon in the core chart and then immediately shifted their attention to the no-core chart area after starting the search. It is important to stress that the user’s cognitive flow in the dashboard interface is closely related to the cognitive flow, unlike the complexity perception process for images or graphics. Therefore, we obtained that layout order consists of the overall visual order (symmetry and unity) and the location of the core chart (browsing order). This can be explained by the principles of visual perception, where humans are accustomed to browsing interfaces from left to right [90]. Consequently, it seems reasonable to assume that when the core chart is in the left-central position, it is more likely to be noticed first, and the browsing order is more in line with the viewing habits of the human eye. In contrast, when the core chart was located in the middle of the interface, the participants started browsing from the center and then spread outwards, resulting in a less organized visual browsing path and longer gaze duration.

In summary, the current study provides evidence that layout order is an important part of dashboard design, and there is an interaction between interface complexity and layout order, where high-level layout order can mitigate the negative effects of complexity and contribute to users’ visual behavior. Moreover, the position of the core chart within the layout order affects the interface complexity. Additionally, the results support the argument that the interface complexity of the dashboard is not an absolute property based on the number of visual elements but rather it is a relative property affected by the degree of visual symmetry and visual unity of the layout form and the position of the core chart.

Besides contributing to complexity theory research, our study provides practical insights for improving dashboard interface design. While some useful strategies have been proposed to reduce the impact of interface complexity [79], these approaches tend to reduce the number of information elements in the interface, which can affect the user’s task completion rate. Therefore, a key challenge in dashboard design is to reduce the impact of complexity without compromising the amount of information in the interface. Our study experimentally confirms that a high-level layout order significantly mitigates the impact of complexity compared to a low-order condition. Thus, layout order should be the focus of dashboard interface design, and designers should adopt the design principle of “order is more” in addition to “less is more”. Furthermore, our experimental results demonstrated the advantages of the interface layout with the core chart in the left-center position. These findings can be directly applied to dashboard-related interface design, especially to complex information interfaces that contain multiple windows or charts. This research can provide valuable inspiration and design guidance for improving interface visual search efficiency and overall system performance.

This research has some limitations. First, the lack of clear design criteria for dashboard interface layouts has led to a wide variety of dashboard interface layouts in practical applications. Although we experimentally manipulated the level of layout order through a simplified abstraction of dashboard interfaces, subjective ratings, and symmetry and unity calculations, and then generated a series of interface materials, there were still some potential confounding factors, such as inconsistencies in the size of the charts, the aspect ratios of non-core charts, and the proportion of white space in the interface, which may impact the layout order. Second, to exclude other confounding factors and manipulate the level of layout order in the stimuli, we simplified the amount of information in the dashboard interface by using icons as a search target. However, the chart information and search targets are more complex in the actual interface, involving multiple forms of coding such as color, text, and animation. For example, previous studies have shown that color has effects on complexity [91] and on user experience [92]. The types of tasks are also not limited to searching for the same target, but also include more difficult data analysis and decision-making judgements. The redundancy of multiple identical targets in the current experimental task may also distract visual attention and affect visual search behavior. Third, the limitations in functionality of the eye tracker we used restricted our ability to obtain temporal and spatial data for the AOI. Therefore, based on the existing research results, future research could further explore the effects of chart size, aspect ratios, the proportion of white space, color, text, and animation on layout order and examine the variability under different task types and the differences in visual attention over space and time.

## 6. Conclusions

This study aimed to investigate the impact of layout order on interface complexity based on eye-tracking techniques. We proposed a concept of “layout order”, which consists of the overall visual order and the location of the core charts. Through the simplified abstraction of dashboard interfaces, subjective ratings, and calculations on symmetry and unity, we succeeded in manipulating the levels of complexity and layout order of the interface materials. Our findings demonstrate that the interface complexity of the dashboard is a relative property affected by the degree of visual symmetry and visual unity of the layout form and the position of the core chart. Specifically, Experiment 1 revealed that there was a significant interaction between interface complexity and layout order, where high-level layout order can mitigate the negative effects of complexity, which supports H1 and H2. Experiment 2 further explored the position of the core chart within the layout order and proved that users perform better when the core chart is positioned on the left. Experiment 2 confirmed that the position of the core chart plays a crucial role in users’ visual search behavior and that the optimal layout order of the dashboard is to place the core chart on the left-center with a partial symmetry of the no-core chart areas. This provided additional support for H3. Our study highlights the effectiveness of eye-tracking techniques in user interface design research and provides valuable insights for dashboard interface design to meet usability needs.

## Figures and Tables

**Table 1:** The calculation measures symmetry and unity.

Factor		Formula
Symmetry	Symmetry is axial duplication: a unit on one side of the center line is exactly replicated on the other side.	$SYM=1-\frac{\left|{SYM}_{vertical}|+|{SYM}_{horizontal}\right|}{2}\in [0,1]$ SYM vertical and SYM horizontal are, respectively, the vertical and horizontal symmetries with ${SYM}_{vertical}=\frac{\begin{array}{c} |{X\prime }_{UL}-{X\prime }_{UR}|+|{X\prime }_{LL}-{X\prime }_{LR}|+|{Y\prime }_{UL}-{Y\prime }_{UR}|+|{Y\prime }_{LL}-{Y\prime }_{LR}|+\\ |{H\prime }_{UL}-{H\prime }_{UR}|+|{H\prime }_{LL}-{H\prime }_{LR}|+|{B\prime }_{UL}-{B\prime }_{UR}|+|{B\prime }_{LL}-{B\prime }_{LR}|+\\ {|\theta \prime }_{UL}-{\theta \prime }_{UR}|+|{\theta \prime }_{LL}-{\theta \prime }_{LR}|+|{R\prime }_{UL}-{R\prime }_{UR}|+|{R\prime }_{LL}-{R\prime }_{LR}| \end{array}}{12}$ ${SYM}_{vertical}=\frac{\begin{array}{c} |{X\prime }_{UL}-{X\prime }_{LL}|+|{X\prime }_{UR}-{X\prime }_{LR}|+|{Y\prime }_{UL}-{Y\prime }_{LL}|+|{Y\prime }_{UR}-{Y\prime }_{LR}|+\\ |{H\prime }_{UL}-{H\prime }_{UR}|+|{H\prime }_{LL}-{H\prime }_{LR}|+|{B\prime }_{UL}-{B\prime }_{LL}|+|{B\prime }_{UR}-{B\prime }_{LR}|+\\ {|\theta \prime }_{UL}-{\theta \prime }_{LL}|+|{\theta \prime }_{UR}-{\theta \prime }_{LR}|+|{R\prime }_{UL}-{R\prime }_{LL}|+|{R\prime }_{UR}-{R\prime }_{LR}| \end{array}}{12}$ ${X\prime }_{j}{,Y\prime }_{j}{,H\prime }_{j},{B\prime }_{j},{\theta \prime }_{j}$$,\;\mathrm{and}\;{R\prime }_{j}$ are, respectively, the normalized values of $X_{j}=\sum_{i}^{n_{j}}{|x}_{ij}-x_{c}|\;\;\;j=UL,UR,LL,LR$ $Y_{j}=\sum_{i}^{n_{j}}{|y}_{ij}-y_{c}|\;\;\;j=UL,UR,LL,LR$ $H_{j}=\sum_{i}^{n_{j}}h_{ij}\;\;\;j=UL,UR,LL,LR$ $B_{j}=\sum_{i}^{n_{j}}b_{ij}\;\;\;j=UL,UR,LL,LR$ $\theta_{j}=\sum_{i}^{n_{j}}|\frac{y_{ij}-y_{c}}{x_{ij}-x_{c}}|\;\;\;j=UL,UR,LL,LR$ $R_{j}=\sum_{i}^{n_{j}}\sqrt{{\left(x_{ij}-x_{c}\right)}^{2}+{\left(y_{ij}-y_{c}\right)}^{2}}\;\;\;j=UL,UR,LL,LR$ where *UL*, *UR*, *LL*, and *LR* stand for upper-left, upper-right, lower-left, and lower-right, respectively; *X_j_* is the total x-distance of quadrant j, *Y_j_* is the total y-distance, *H_j_* is the total height, *B_j_* is the total width, j is the total angle, and *R_j_* is the total distance; (*x_ij_*; *y_ij_*) and (*x_c_*; *y_c_*) are the coordinates of the centers of object i on quadrant j and the frame; *b_ij_* and *h_ij_* are the width and height of the object; and *n_j_* is the total number of objects on the quadrant.
Unity	Unity is coherence, a totality of elements that is visually all one piece.	$UN=\frac{\left|{UN}_{form}+{UN}_{space}\right|}{2}\in [0,1]$ UM form is the extent to which the objects are related in size with ${UN}_{form}=1-\frac{n_{size}-1}{N}$ and UM space is a relative measure of the space between groups and that of the margins with ${UN}_{space}=1-\frac{a_{layout}-\sum_{i}^{n}\left(w_{i}\times h_{i}\right)}{width\times height-\sum_{i}^{n}\left(w_{i}\times h_{i}\right)}$ where a_i_, a_ayout_, and a_frame_ are the areas of object i, the layout, and the frame, respectively; n_size_ is the number of sizes used; and n is the number of objects on the frame.

**Table 2:** Symmetry and unity calculation results based on the esthetic model.

Factor		Symmetry	Unity	Total
Low complexity	High-level order	1	0.91	1.91
	Mid-level order	0.96	0.52	1.48
	Low-level-order	0.9	0.38	1.28
Medium complexity	High-level order	1	0.9	1.9
	Mid-level order	0.96	0.47	1.43
	Low-level-order	0.92	0.33	1.25
High complexity	High-level order	1	0.89	1.89
	Mid-level order	0.89	0.66	1.55
	Low-level-order	0.8	0.50	1.3

**Table 3:** The mean, SD, and standard error of eye movement data in TFC and TGD.

Variables		TFC			TGD		
Complexity	Order	Mean	SD	SE	Mean	SD	SE
Low	disordered	5.170	2.111	0.08	0.874	0506	0.020
	ordered	4.286	1.297	0.10	0.748	0.375	0.030
Medium	disordered	10.158	4.201	0.17	1.755	0.804	0.032
	ordered	8.899	3.161	0.356	1.561	0.708	0.080
High	disordered	14.048	5.951	0.239	2.543	1.090	0.044
	ordered	12.257	7.082	0.691	2.196	1.224	0.120

**Table 4:** Comparison of scanning paths between high-level and low-level layout order interfaces.

	High-Level Order Interfaces	Low-Level Order Interfaces
Typical participant’s scanning path	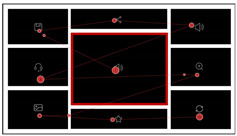	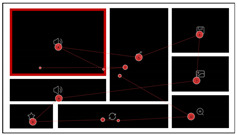
The overlapped scanning paths of all participants	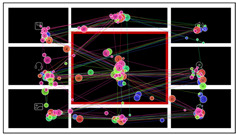	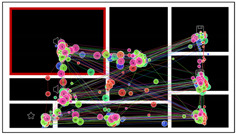

**Table 5:** Comparison of scanning paths between mid-central and high-order and left-central and mid-order interfaces.

	Mid-Central and High-Order Interfaces	Left-Central and Mid-Order Interfaces
Typical participant scanning path	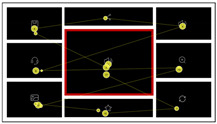	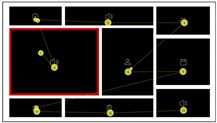
The overlapped saccade tracks	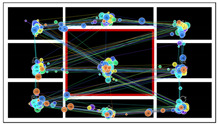	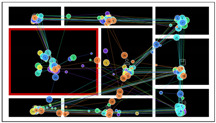

## Data Availability

Data are contained within the article.
